# Next generation multi-scale biophysical characterization of high precision cancer particle radiotherapy using clinical proton, helium-, carbon- and oxygen ion beams

**DOI:** 10.18632/oncotarget.10996

**Published:** 2016-08-01

**Authors:** Ivana Dokic, Andrea Mairani, Martin Niklas, Ferdinand Zimmermann, Naved Chaudhri, Damir Krunic, Thomas Tessonnier, Alfredo Ferrari, Katia Parodi, Oliver Jäkel, Jürgen Debus, Thomas Haberer, Amir Abdollahi

**Affiliations:** ^1^ German Cancer Consortium (DKTK), Translational Radiation Oncology, National Center for Tumor Diseases (NCT), German Cancer Research Center (DKFZ), Heidelberg, Germany; ^2^ Heidelberg Institute of Radiation Oncology (HIRO), National Center for Radiation Research in Oncology (NCRO), Heidelberg, Germany; ^3^ Heidelberg Ion-Beam Therapy Center (HIT), Heidelberg, Germany; ^4^ Department of Radiation Oncology, Heidelberg University Hospital, Heidelberg, Germany; ^5^ National Center for Oncological Hadrontherapy (CNAO), Pavia, Italy; ^6^ Light Microscopy Facility, German Cancer Research Center, Heidelberg, Germany; ^7^ Department of Medical Physics, Faculty of Physics, Ludwig-Maximilians-Universität München, Munich, Germany; ^8^ European Organization for Nuclear Research CERN, Geneva, Switzerland; ^9^ Division of Medical Physics in Radiation Oncology, German Cancer Research Center, Heidelberg, Germany

**Keywords:** radiobiology, DNA-double strand breakages, complex DNA damage, monte carlo simulations, biophysical hybrid detectors

## Abstract

The growing number of particle therapy facilities worldwide landmarks a novel era of precision oncology. Implementation of robust biophysical readouts is urgently needed to assess the efficacy of different radiation qualities. This is the first report on biophysical evaluation of *Monte Carlo* simulated predictive models of prescribed dose for four particle qualities i.e., proton, helium-, carbon- or oxygen ions using raster-scanning technology and clinical therapy settings at HIT. A high level of agreement was found between the *in silico* simulations, the physical dosimetry and the clonogenic tumor cell survival. The cell fluorescence ion track hybrid detector (*Cell-Fit-HD*) technology was employed to detect particle traverse per cell nucleus. Across a panel of radiobiological surrogates studied such as late ROS accumulation and apoptosis (caspase 3/7 activation), the relative biological effectiveness (RBE) chiefly correlated with the radiation species-specific spatio-temporal pattern of DNA double strand break (DSB) formation and repair kinetic. The size and the number of residual nuclear γ-H2AX foci increased as a function of linear energy transfer (LET) and RBE, reminiscent of enhanced DNA-damage complexity and accumulation of non-repairable DSB. These data confirm the high relevance of complex DSB formation as a central determinant of cell fate and reliable biological surrogates for cell survival/RBE. The multi-scale simulation, physical and radiobiological characterization of novel clinical quality beams presented here constitutes a first step towards development of high precision biologically individualized radiotherapy.

## INTRODUCTION

More than 50% of cancer patients receive radiotherapy in their course of disease. Hence, radiotherapy is a cornerstone in multimodal management of cancer. The emergence of particle therapy constitutes the latest technological development towards achieving a conformal irradiation of tumor while sparing normal tissue [1]. The worldwide growing number of proton (> 50 centers, > 100,000 patients treated) and carbon ion facilities (8 centers, > 12,000 patient treated) underscores the relevance and high expectations attributed to this promising strategy towards development of modern precision oncology [1] (http://www.ptcog.ch/). However, reliable and robust biophysical benchmarking experiments are needed to validate the quality of the beams on the routine basis in and across the facilities.

In addition to precision and physical characteristics, specific biological properties of different ion species (e.g. helium-, carbon-, neon-, silicon- or argon ions) to circumvent tumor radioresistance mechanisms, were postulated by pioneering experiments conducted at Lawrence Berkeley Laboratory [2]. Several decades later, after implementation of cutting-edge technological approaches, such as raster scanning method, the Heidelberg Ion-Beam therapy Center (HIT) now provides the opportunity for a back to back comparison of four promising radiation qualities, i.e., proton (^1^H), helium- (^4^H), carbon- (^12^C) and oxygen ions (^16^O). Here we report an approach to define a radiobiological quality assurance strategy and comparative studies across these four radiation qualities. Based on currently mostly advanced physical planning models *in-silico*, Monte Carlo (MC) simulations [3] of prescribed dose for each ion source were conducted. The quality control further consisted of a tissue equivalent water phantom-based physical dosimetry, which is currently employed for dose verification of patient plans [4]. The prescribed physical dose was then correlated with clonogenic cell survival as the gold standard radiobiological readout [5].

Controversies exist on the impact of enumerating total vs. residual radiation induced DNA-double strand breakages determined by immunostaining strategies such as nuclear phosphorylated histone H2AX (γ-H2AX) foci as radiobiological surrogates [6–8]. Hence, we evaluated the γ-H2AX foci size and dynamic (initial vs. persistent) as a function of dose, radiation source and cell survival. Finally, a recently engineered cell-fluorescent ion track hybrid detector (Cell-Fit-HD) technology was employed to detect the number of primary particle hits per cell nucleus in correlation with *in-silico* MC simulation. Together, we provide a reliable and robust strategy for radiobiological quality control (QC) as a benchmark for clinical particle facilities as well as centers working with high-energy particles and space radiation.

## RESULTS

### Comparative radiotherapy plan simulation in a cancer patient

**Figure 1:**
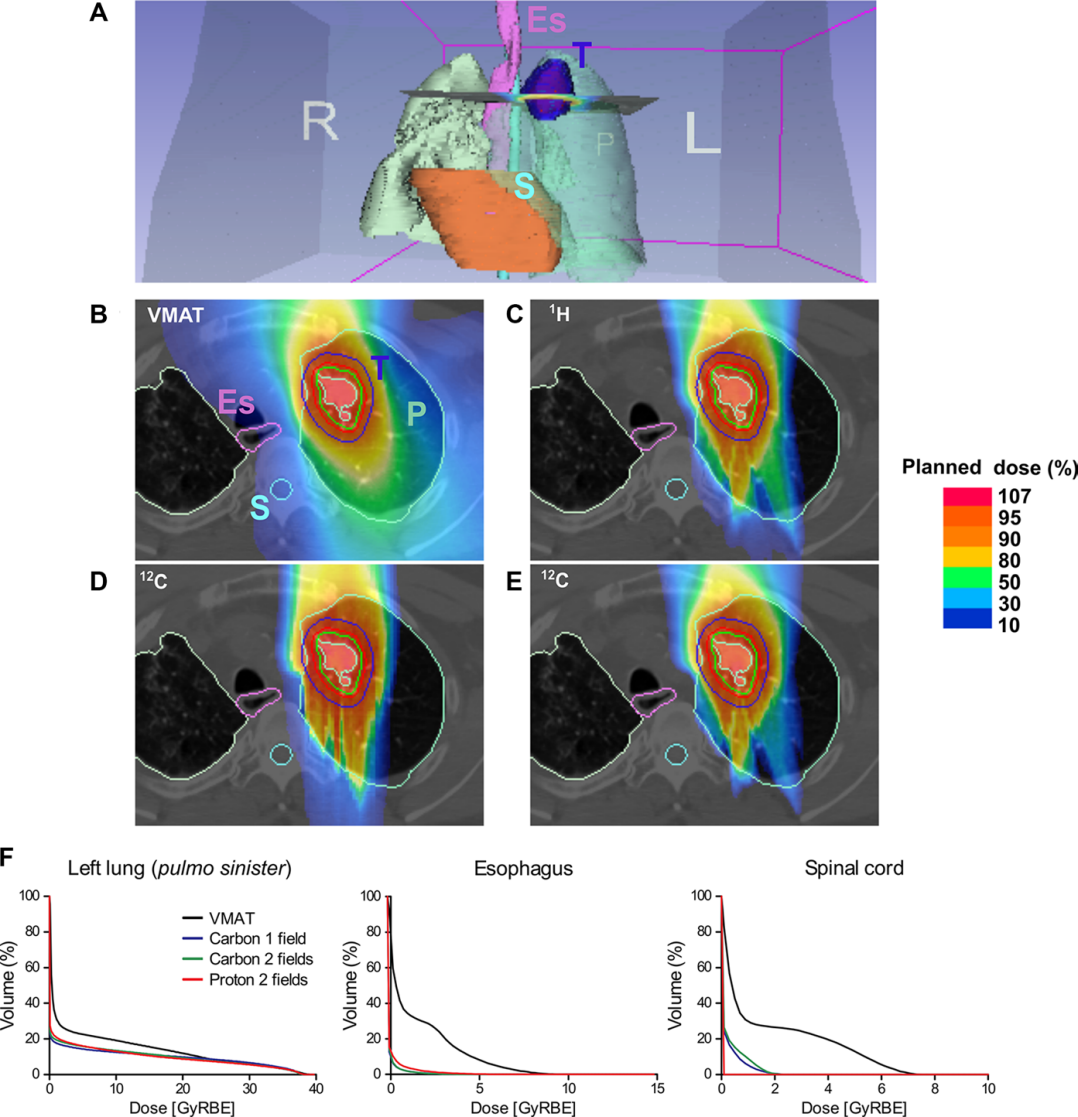
Comparative simulation of radiotherapy plans in a lung cancer patient (**A**) Overview; anatomical tumor localization. Dose distribution of (**B**) reference (photon) irradiation using Volumetric Intensity Modulated Arc Therapy (VMAT); raster scanning proton therapy with (**C**) two fields, (**D**) carbon therapy with a single field, (**E**) carbon therapy with two fields. A relatively larger volume including esophagus (Es) and spinal cord (S) receives radiotherapy after VMAT compared to particle therapies. (**F**) Dose-volume histograms (DVH) confirm superior sparing of different organs at risk (OAR) using the particle therapy plans compared to VMAT. P: lung (*Pulmo*), E: esophagus (pink contour), S: spinal cord (light blue contour), T: tumor (dark blue contour), GTV (red contour), CTV (green contour), PTV (light green contour). L: left; R: right. Percentage of a planned dose is displayed using a color bar.

A prototypic indication for particle therapy is the clinical situation that requires very high precision i.e. administration of carbon ions for intracranial tumors (chordoma) close to radiosensitive brain-stem [9]. Another example would be application of protons in pediatric tumors such as carnio-spinal (neuro-axis) irradiation of medulloblastoma where maximal preservation of normal tissue, low-dose volume, is attempted to reduce normal tissue complication probability (NTCP) and late radiation effects such as secondary cancers. We aimed to demonstrate comparative plans for a clinical case, where radiobiology, in addition to precision, rationalized usage of carbon ions. (Figure 1A–1E) demonstrates alternative treatment plans. Calculated dose-volume histograms (DVH) for organs at risk (OAR) are given in Figure 1F. DVH for three main radiotherapy target volumes (planning target volume-PTV, clinical target volume – CTV, and gross tumor volume –GTV) are shown in Supplementary Figure S1.

The recurrent small cell lung cancer tumor volume post conventional radiotherapy delivered by helical tomotherapy was delineated in the CT-scan. After three lines of chemotherapy and previous radiation, the patient was systemically controlled and was in a relatively good health status. The clinical challenge was to re-irradiate this radioresistant tumor and simultaneously preserve OAR such as the esophagus and the lung. The probability of radiation induced esophagitis, strictures/perforation, pneumonitis and lung fibrosis directly correlates with the irradiated dose and volume. Simulation of dose distribution using an advanced photon irradiation method, Volumetric Intensity Modulated Arc Therapy (VMAT) [10] was compared to the two currently clinically commissioned therapy options, i.e., proton and carbon ion with either a single or two fields (Figure 1). Particle irradiation of lung tumors is challenging due to the anatomy and breathing (motion) effects leading to beam scattering or variability in deposition of energy in tissue depth (over-/under-shooting phenomenon) that occurs as a function of tissue density dependent slowdown of particles i.e., bone (rib) vs. intercostal soft tissue vs. lung parenchyma vs. air. Nonetheless, as dose-volume histograms indicate, representing the corresponding dose per irradiated volume for each plan, most OAR such as the esophagus, lung and spinal cord receive a substantially lower dose with particles vs. photon irradiation. Based on tumor refractoriness to conventional radiotherapy and the hypoxic tumor phenotype we decided to prescribe carbon ion irradiation. With commissioning of the gantry at HIT (fall 2012) this was the first lung cancer patient to be treated using a single field in vertical (0°) anterior-posterior (AP) beam direction by raster scanning carbon ions. The patient well tolerated the therapy and the recurrent tumor regressed within weeks after irradiation.

### Simulation and physical dosimetry

**Figure 2:**
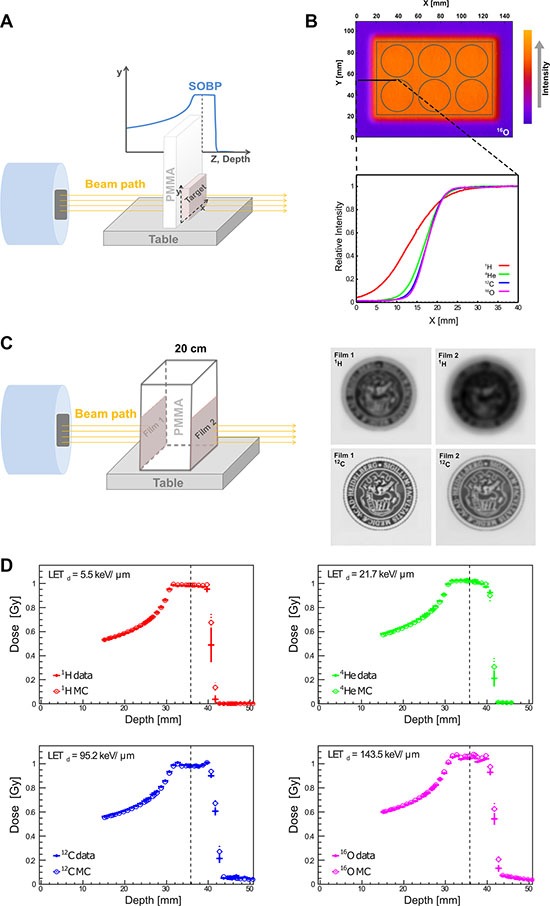
Physical depth-dose distribution and lateral scattering (**A**) Schematic presentation of irradiation setup. To mimic the clinical situation of tumor treatment at a certain tissue depth, PMMA was employed as water/tissue density equivalent and placed in front of the target (cell culture plate). (**B**) The corresponding intensity distribution of oxygen beam for a six well plate is presented according to the Fire Lookup Table, where the lowest intensity is presented in purple color, and highest intensity in orange color. The normalized lateral intensity distribution (0–40 mm along X-axis, black solid line) of all four investigated particles is shown (bottom). As the beam mass increases, the steepness of the lateral distribution increases, due to the reduced scattering for heavier ions. (**C**) Schematic presentation of lateral scattering in proton and carbon ion beams. Left panel presents the irradiation setup used to demonstrate lateral scattering for proton and carbon beams. Right panel are the scanned images of irradiated dosimetric films. (**D**) High correlation between the MC simulation (empty circles) vs. physical dosimetry (data, filled circles) of depth-dose distributions in water phantom for all four investigated particles. The vertical dashed lines mark the reference depth where the cells were located for subsequent biophysical readouts and the corresponding LET is provided for each particle: proton (^1^H), helium (^4^He), carbon (^12^C), oxygen (^16^O) irradiation beams.

To explore the relative biological effects across different radiation qualities compared to reference photon irradiation, a clinical-like experimental setting was designed. Tumor cells were placed at the mid spread out Bragg peak (SOBP), corresponding to a tissue depth of 3.5 cm using fixed horizontal clinical beams available at Heidelberg Ion Therapy Center, as schematically shown in Figure 2A. As the first step for quality assurance of irradiation plans and beam delivery in the target, we analyzed and compared lateral dose distributions as shown in Figure 2B. Figure 2B displays a representative ^16^O plan, where normalized radiation dose intensity levels are superimposed on the geometry of the irradiated 6-well plates. Comparing lateral intensity fall-offs at the edge of the 6-well plates (Figure 2B bottom) in terms of 90%–10% variation, i.e. the difference between the distances needed to reach the 90% and 10% of the intensity, they increase as the mass of the beam particle decrease. The latter is mainly due to the scattering phenomenon (see Figure 2C), and also due to the limited effect of nuclear secondaries in the studied set-up. The measured fall-off values are: 17.5 mm for ^1^H, 9.7 mm for ^4^He, 8.1 for ^12^C and 7.7 mm for ^16^O. Figure 2C represents a schematic explanation of lateral scattering in proton and carbon ion beams. Two films (Film 1 and Film 2) were positioned at the entrance and at 20 cm depth and irradiated with proton (220 MeV) and carbon ions (380 MeV/u). The 20 cm depth has been selected as representative of deep-seated tumor in the patients. The images corresponding to proton and carbon ions at 20 cm depth are more blurred compared to the ones at the entrance. This phenomenon is due to the beam interaction with the matter in terms of multiple Coulomb scattering (MCS) and productions of nuclear secondaries (in case of carbon ion beams). Comparing proton and carbon ion results, sharper contrast images have been obtained in case of carbon ions. The reason for this is that the MCS effect decreases with the increase of the mass of the beam.

Next, depth-dose distributions for all four available radiation qualities were measured using water phantom as tissue equivalent and experimental data were compared with MC simulations. This is the first report on physical QC of raster scanning oxygen and helium beams at HIT. Our data indicate a high level of agreement for all investigated particles as shown in Figure 2D. The mean and standard deviation of the absolute percentage difference between dosimetric data and MC results for each irradiation in the relevant depth interval (before 4 cm water equivalent depth where the distal fall-off starts) is: 0.7% ± 0.6% for protons, 1.1% ± 0.6% for helium-, 1.3% ± 0.6% for carbon- and 1.2% ± 0.8% for oxygen ions.

### Relative biological effectiveness of different radiation beam qualities

**Figure 3:**
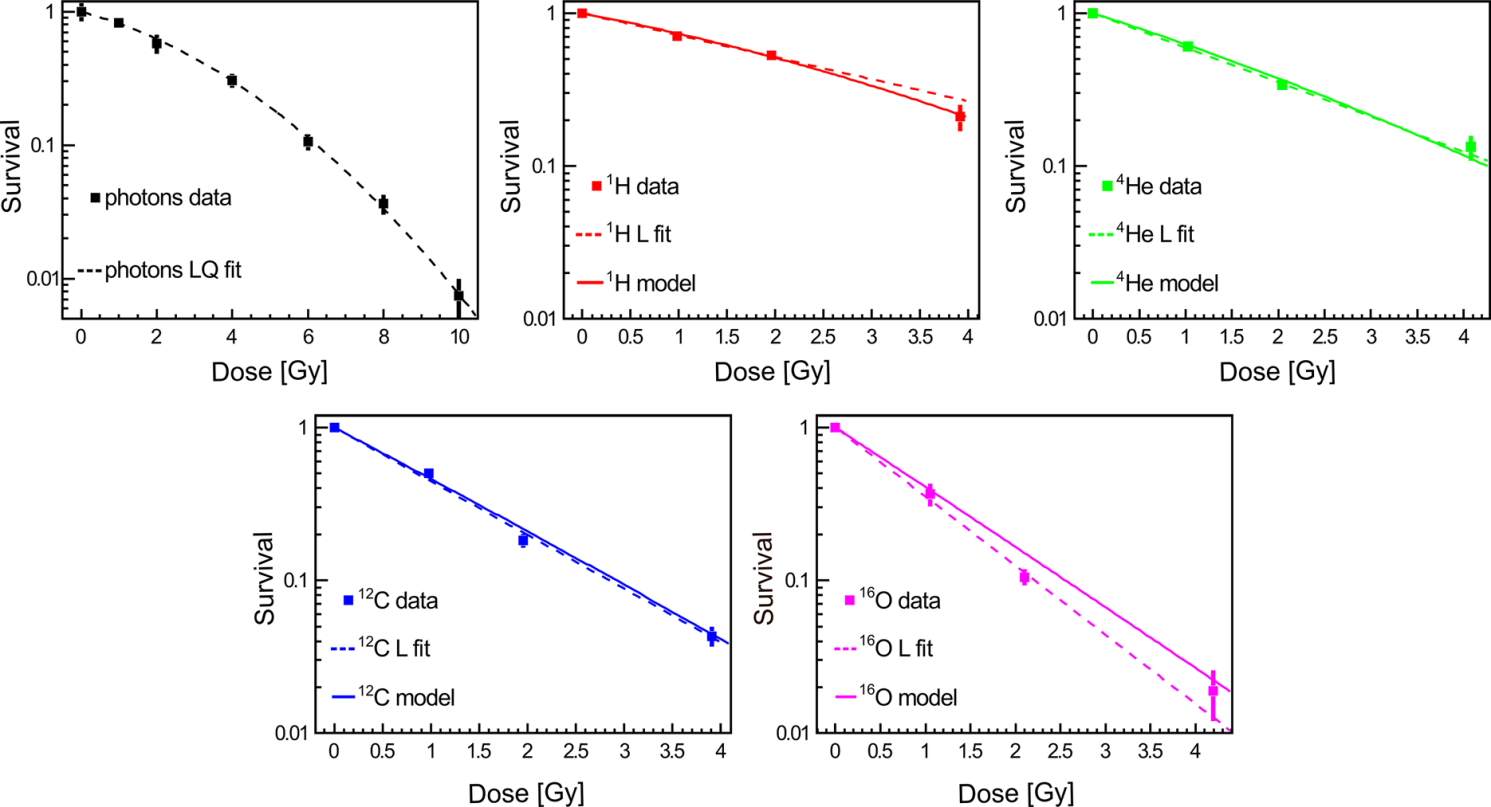
Clonogenic cell survival as a function of dose and radiation quality Data represent mean ± SEM of three independent experiments, each performed in triplicates. The dashed lines represent the linear-quadratic (LQ) or linear (L) fits of experimental data that strongly correlated with model simulations.

Clonogenic cell survival assay is considered as a gold standard method for studying cellular sensitivity to irradiation. This is in line with our observation evaluating a panel of radiobiological surrogates for relative biological effectiveness (RBE) in this study such as late reactive oxygen species (ROS) accumulation and apoptosis (see Supplemental Material). To assess the effect of different irradiation beams on cell survival, A549 cells were irradiated with photons (as the reference radiation treatment), ^1^H, ^4^He, ^12^C, ^16^Oions at different clinically relevant doses (Figure 3). Irradiation of A549 cells revealed higher RBE values for higher dose-averaged LET (LETd). The clonogenic survival data have been fitted (dashed lines in Figure 3) with the LQ model for photons while for particle beams a simple linear (L) dependence was statistically preferable in describing the experimental data in the examined dose range. Moreover, predictions of the cell survival data have been performed applying the MC code FLUKA and HIT re-implementation of the GSI LEM-IV model (solid lines in Figure 3). The experimentally obtained and model-based values for RBE at 30% survival (RBE_30_) are reported in Table 1 and describe the inverse correlation between RBE_30_ and LETd. The MC predictions of the RBE_30_ match the experimentally derived data.

**Table 1:** Fit parameters and relative biological effectiveness

Beam modality	α [Gy^−1^]	β [Gy^−2^]	RBE30	RBE_30_ - MC
Photons	0.173 ± 0.026	0.032 ± 0.004		
Protons	0.332 ± 0.011		1.1 ± 0.2	1.2
Helium ions	0. 521 ± 0.023		1.7 ± 0.3	1.7
Carbon ions	0.809 ± 0.047		2.7 ± 0.5	2.6
Oxygen ions	1.041 ± 0.086		3.5 ± 0.6	3.0

For each beam modality, the fits parameters of the LQ (photons) or L (particles) models and the resulting RBE_30_ (relative biological effectiveness for 30% survival) and corresponding standard deviations are reported in columns from two to four. MC-calculated RBE30 is reported in the last column.

### Biophysical QC via Cell-FIT HD

**Figure 4:**
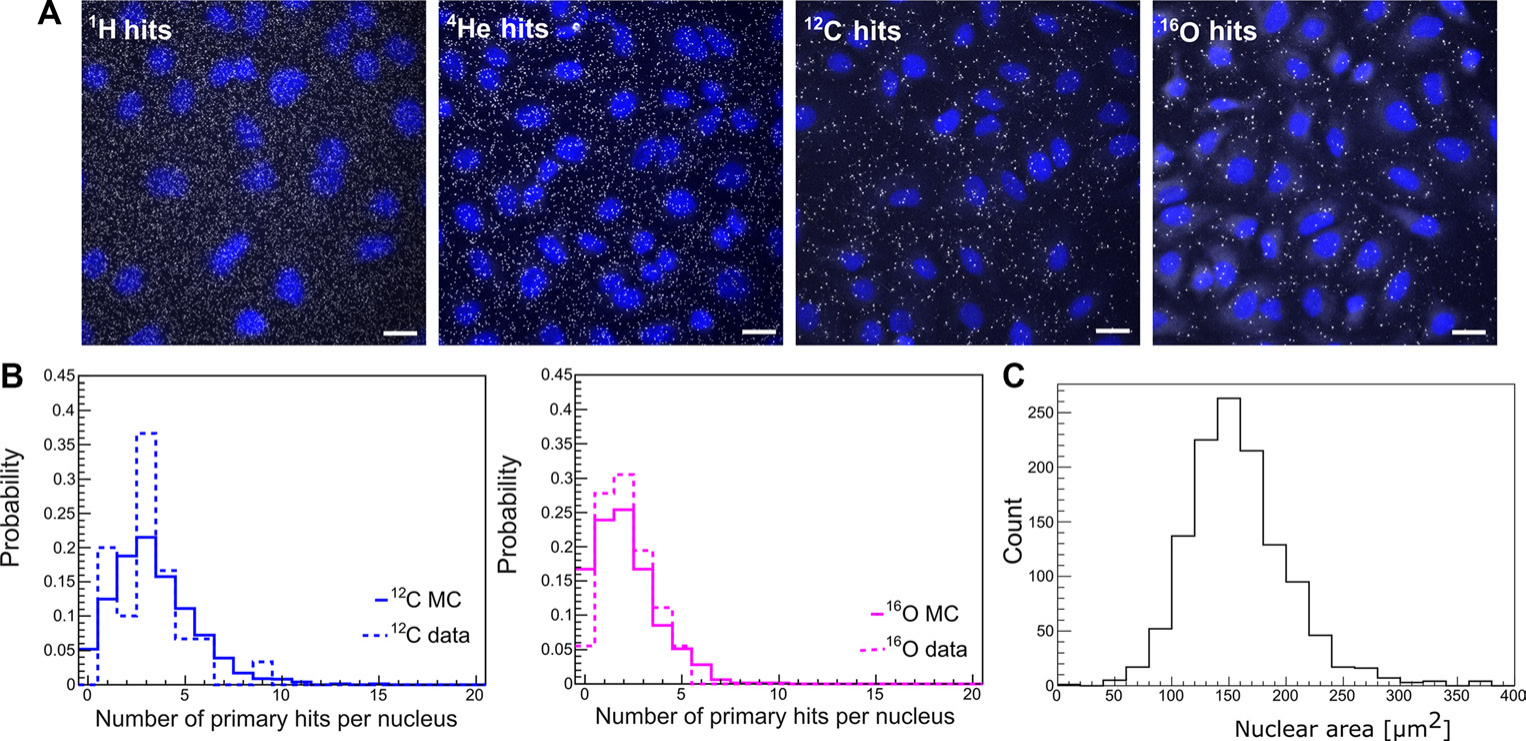
Particle hit per cell nucleus Cell-Fit-HD was employed to detect the number of primary ion hits per cell nucleus. (**A**) White dots represent particle traversals and blue areas the cell nuclei (DAPI stained). Scale bar: 20 μm. (**B**) A high correlation between experimental (3.2 ± 0.3 for ^12^C-beams and 2.2 ± 0.2 for ^16^O-beams) vs. simulation (3.4 for ^12^C-beams and 2.1 for ^16^O-beams) based nuclear hit distribution was found. Y-axis represents the probability of particle hits per nucleus. (**C**) Nuclear area size distribution. Images of DAPI-stained nuclei were obtained. To measure nuclear area the Z-stack images of DAPI staining were background subtracted using ImageJ's Rolling ball radius. The images were further maximum Z-projected and segmented using Median filter to more precisely define the nuclear border. The images were thresholded and nuclear area was finally measured using the Analyze Particles tools. All the image processing was performed automatically using the ImageJ macro with constant settings (*n* = 1239).

The recently introduced Cell FIT-HD technology allows for simultaneous visualization of cells (nuclei) and the particle beam traversal (hits per cell nucleus) identified as fluorescence spots in the physical detector component (FNTD, Figure 4A). Each spot is characterized by an intensity that corresponds to the physical energy deposition. These intensities can be separated in two populations: higher intensities define primary particles and heavy projectile-like particles (high LET particles), and lower intensities define lighter fragments [11]. In here-described clinical settings (mid SOBP), the contribution of primary particles (hits) for cytotoxic effect was more than 90% for ^12^C and ^16^O beams. MC simulations for primary intranuclear hit statistics (Figure 4B) were performed taking into account the measured nuclear area distribution (Figure 4C), i.e., randomly sampling the area of cellular nuclei from the measured values and applying the Poisson statistics. The Cell-Fit-HD measured mean (±SEM) of the intranuclear hits were 3.2 ± 0.3 and 2.2 ± 0.2 for carbon ion and oxygen ion beams, respectively (Figure 4B). The corresponding MC calculated values were 3.4 and 2.1 for ^12^C and ^16^O, respectively. Together, the comparison of simulated statistics vs. experimental Cell-Fit-HD measures was in good agreement for the number of primary particle hits per cell nucleus.

**Figure 5:**
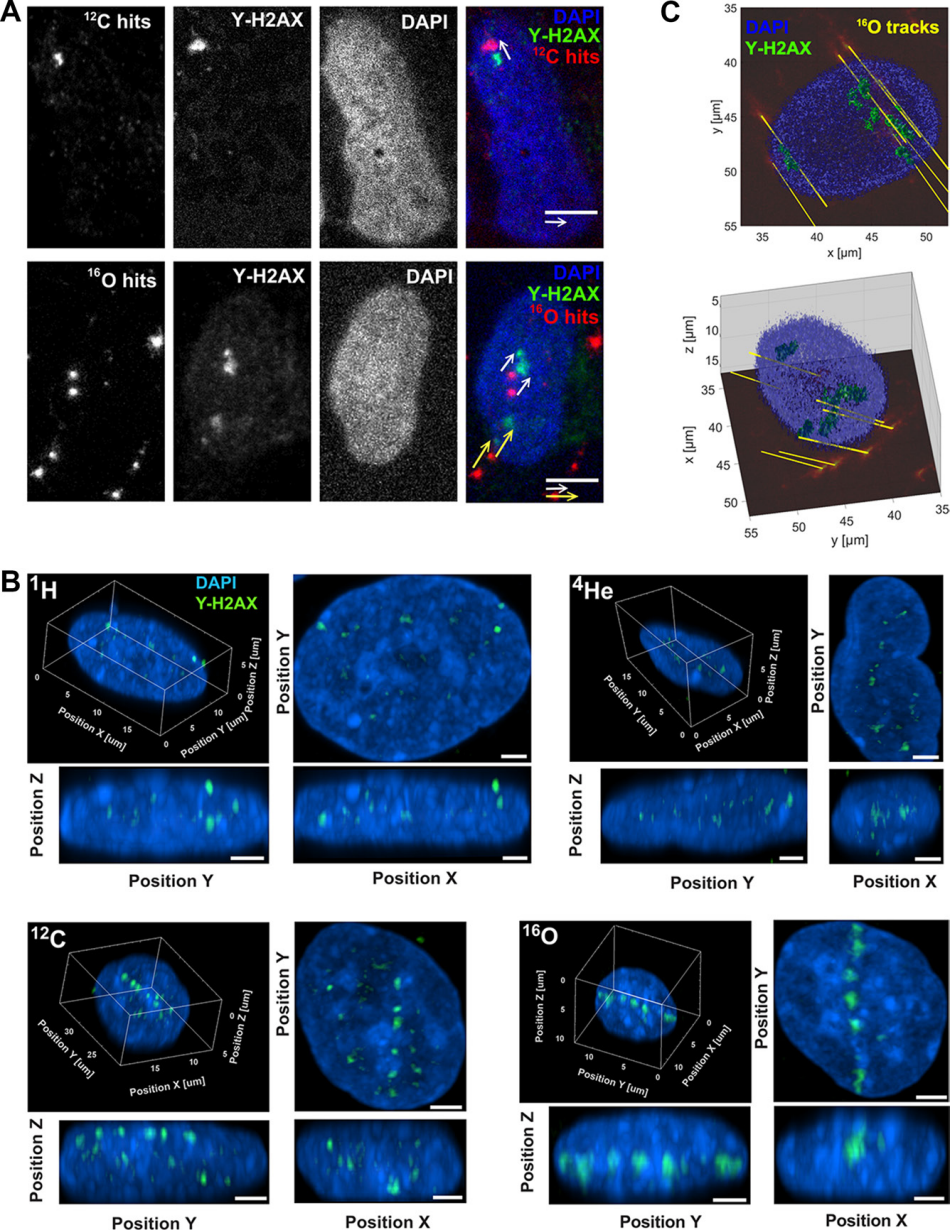
Correlation of particle hits and DNA-damage (**A**) Representative ^16^O and ^12^C particle traversals (physical compartment, FNTD, red pseudocolor) projection to the corresponding nuclear (DAPI, blue) DNA-damage (DSB) marked by γ-H2AX foci (green) in the biological compartment. The distances (~ 3 μm) between the hits and foci are a consequence of a movement of the cell nuclei. Scale bar: 5 μm. (**B**) 3D visualization of γ-H2AX foci (green) formation along particle traversals (trajectories) more clearly visible after high LET irradiation (carbon and oxygen beams). Scale bar: 2 μm. (**C**) 3D reconstruction of γ-H2AX foci and ^16^O ion traversal utilizing Cell-Fit-HD (^16^O, tilted irradiation 45°, 0.25 Gy). Ion traversals were traced over 19 consecutive FNTD imaging planes (total range in z: 57 μm).

Interaction between particles and DNA may lead to formation of difficult to repair DNA damage, such as complex DSB, which are considered as hallmarks of radiation induced lethal effects [12, 13]. We analyzed the spatial nuclear distribution of DSB foci and particle tracks patterns using the phosphorylated histone H2A variant (γ-H2AX) as the surrogate marker. Intranuclear particle traversals of ^12^C and ^16^O detected on the FNTD coincided with the appearance of corresponding nuclear γ-H2AX foci on the Cell-Fit-HD (Figure 5A). The slope of the translation vector equals for all track-focus pairs. 3D morphology of irradiation induced γ-H2AX foci in the nuclei are shown in Figure 5B. Cell-Fit-HD was also used to reconstruct and visualize ^16^O primary ions traversing the nucleus in 3D, as well corresponding γ-H2AX foci (Figure 5C).

### Biological radiodosimetry via quantitative double-strand breaks

**Figure 6:**
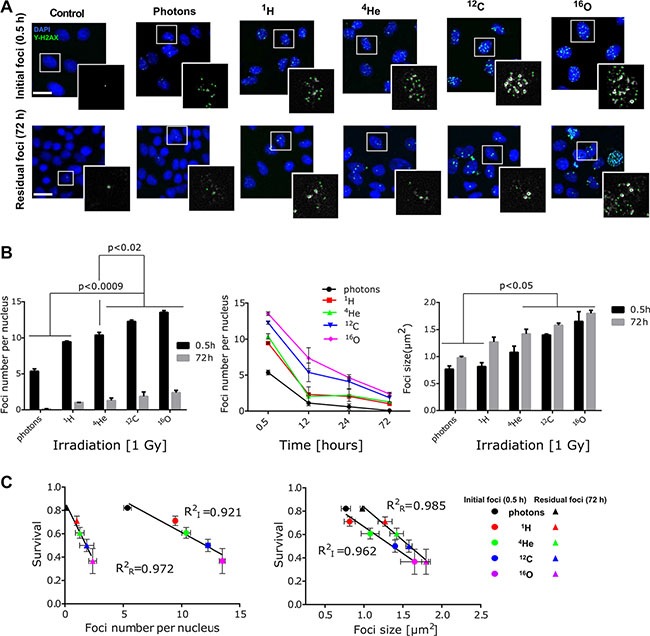
Evaluating DSB number, size and repair kinetic as biodosimeter (**A**) Representative images of γ-H2AX foci (green) initial (0.5 h) and residual (72 h) after 1 Gy (physical dose) irradiation of all five-radiation qualities. Cell nuclei were counterstained with DAPI (blue). Selected nuclei (in white squares) are magnified to show the foci included for quantitative analysis (marked with green dots). Scale bar: 20 μm. (**B**) Formation γ-H2AX foci (0.5 h) and repair kinetic was longitudinally investigated up to 72 h post irradiation. ^12^C and ^16^O ion beams induced significantly higher number and size of γ-H2AX foci with slower resolution kinetics, compared to other irradiation beams (photons, ^1^H, ^4^He). Bars represent mean ± SEM of at least two independent experiments. More than 100 nuclei per sample, per experiment were analyzed. (**C**) Correlation of clonogenic survival fractions with foci size and enumeration of total initial (I; 0.5 h) vs. residual (R; 72 h) foci as function of LETd has been found. Pearson correlation statistics for foci count (initial *p* = 0.001, residual *p* = 0.0096); foci size (initial: *p* = 0.0053, residual *p* = 0.0008).

Next we aimed to explore the value of DNA DSB damage characteristics such as foci size, repair kinetics and quantification as biological surrogates for the prescribed dose of different radiation qualities. A panel of cell lines was screened for the presence of background γ-H2AX positive nuclear stains including SSC25, PC3, Caki2, FADU, DU145, A431, CAL27, T98G, BXPC3, Panc1, HCT116, U87, U20S, A375 and A549 cells (ordered according to the background foci content, from highest SSC25 to the lowest A549). Therefore, A549 cell line was selected due to its relatively low background foci levels. Analysis of γ-H2AX foci kinetics and size in A549 cells was conducted using maximum intensity projection (MIP) of a stack in the central region of the nuclei (Figure 6). The images revealed differences in the radiation induced foci (RIF) number and size after 1 Gy physical dose irradiation with different beam qualities (Figure 6A). The analysis indicated that the number of initial RIF at early time point (0.5 hour post-irradiation) was lower in the cells irradiated with low LETd beams (photon, ^1^H, ^4^He) in comparison to the cells irradiated with higher LETd (^12^C and ^16^O). Moreover, irradiation with higher LETd led to the formation of larger RIF (Figure 6B). Temporal analysis of foci at 12, 24 and 72 hour post-irradiation, shows irradiation modality-dependent γ-H2AX resolution kinetics. The results indicate slower DSB repair rates for cells irradiated with high LETd beams (^12^C, ^16^O; 95.2 keV/μm and 143.5 keV/μm, respectively). At 72 h post irradiation in photon-irradiated samples, the majority of the RIF were resolved (97.7%). In contrast, in particle-irradiated cells, potentially irreparable, residual foci, especially after high LETd ^12^C (15.3%) and ^16^O (17.6%) irradiation, were detected (Figure 6B). The residual, persistent RIF were found to be larger in size compared to the initial foci, in particular after high vs. low LETd irradiation.

To further determine the impact of foci size, initial- and residual foci numbers as biodosimeters, correlation analysis with clonogenic cell survival as the current standard were conducted (Figure 6C). Those data indicated that both increased foci number (per nucleus) and size strongly correlates with a reduced cell survival fraction. Calculated coefficient of determination (R^2^) for initial RIF number per nucleus at 0.5 h post-irradiation (R_I_^2^ values) equaled 0.921 whereas for residual RIF (R_R_^2^) it was higher and equaled 0.972. In case of RIF size R_I_^2^ and R_R_^2^ were 0.962 and 0.985, respectively.

## DISCUSSION

Development of novel approaches for biophysical dosimetry and definition of robust benchmarks are needed to ensure the high quality of novel clinically available cancer particle therapies. Towards this goal, we devised a platform consisting of *in silico* simulation of biological experiments and experimental validation of the simulated data. In line with current approach to verify patient radiotherapy plans for proton and carbon ion therapy, we employed physical dosimetry in water phantom and report here successful QC data on the two new particle beams (helium and oxygen) at HIT. We found a high level of agreement between the MC-simulation and the depth- dose distribution assessed by physical dosimetry data for all four evaluated radiation qualities.

Precise dose delivery in tumor mass with a steep dose gradient sparing surrounding normal tissues (organs at risk) is a unique feature of particle therapy [14]. However, biological effectiveness, depth-dose and lateral dose distributions can differ between different particle beams. Based on these differences and tumor characteristics the optimal beam therapy could be individualized for each cancer patient [9, 14]. As shown in Figure 1, heavier particles ensure a higher level of dose conformity, which improves tumor control while sparing normal tissues and organs at risk [15]. For example, in this work, remarkable steep lateral distribution has been found in case of ^4^He beams compared to proton beams strengthening its possible future clinical application, as it was suggested in earlier studies [16, 17]. Helium ions exhibit advantageous physical characteristics: reduced lateral scattering when compared to proton beams [18] and reduced fragmentation tail compared to carbon ion beams [16]. Oxygen beams have an enhanced fragmentation tail but, compared to carbon ions, provide higher linear energy transfer (LET) distribution in the target [19], bringing possible clinical advantages for treating radio-resistant, e.g. hypoxic tumors [20].

Comparing the biological effects of different radiation modalities requires a solid prediction and evaluation of the absorbed dose distribution. To our knowledge, we report here the first successful correlation between the simulation data and the population level statistics of ^12^C and ^16^O ions hits per cellular nucleus using clinical settings and a recently engineered biophysical hybrid detector (Cell-Fit-HD) technology [21]. Of note, the relatively high fluency of particles investigated in patient treatment like- settings limited clear discrimination of single particle traversal and consequently the application of nuclear hit statistics for ^4^He and ^1^H. Perhaps further improvements of the FNTD readout technology will overcome this shortcoming. Alternative strategies may further be utilized such as e.g. extrapolation from lower fluencies or positioning the target in the raising flank rather than middle of the SOBP as recently described for Cell-Fit-HD-based spatial correlation of single ^12^C particle traversals and DNA damage foci [11]. In analogy, we visualized ^16^O ions particles, in clinical settings, traversing the cell nuclei and performed 3D reconstruction of ion traversals and corresponding γ-H2AX foci. The spatial correlation is of high significance as a broad spectrum of sources such as secondary particles, gamma irradiation and superimposition effects may as well lead to appearance of radiation induced foci as double-strand break surrogates. However, readout of the physical dose deposition compartment of Cell-Fit-HD, allows a clear discrimination of primary vs. secondary/lower energy particles and gamma irradiation. Hence, we show that nuclear hit statistics could be precisely investigated using Cell-Fit-HD. Moreover, the spatial localization of particle traverse enables detailed studies of biological consequences of high LET primary irradiation. Our data build on previous seminal work conducted in the area of solid state detectors with ramifications beyond the field of cancer therapy, e.g., may facilitate a better biophysical characterization of space radiation [22, 23].

RBE constitutes an important parameter for dose prescription in particle treatment planning [24, 25]. Therefore, we analyzed RBE for clonogenic survival as a gold standard for evaluation of cellular radiosensitivity. Model predictions performed in this study, i.e. interfacing the MC transport and interaction capability with an external biophysical model [26], correlated well with the experimental clonogenic survival data suggesting reliable RBE estimation for different radiation qualities using our algorithm. This study represents a starting point towards *in silico* (MC-based) prediction of tumor RBE towards individualized selection of particle beam based on patients' radiobiological tumor characteristics [27, 28].

A controversially debated theme in the field of radiobiological dosimetry and RBE assessment is the impact of total number of radiation induced DSB determined by RIF count within minutes after irradiation versus the quantity of the residual RIF hours/days post irradiation on prediction of cellular radiosensitivity [7, 8, 29]. Most commonly used approach for identification of irradiation induced DSB is determination of γ-H2AX RIF [30, 31]. However, the appearance of γ-H2AX positive foci *per se* might not be a consequence of radiation induced DSB. Formation of non-irradiation induced foci is attributed to various molecular processes in the cells such as environmental stress and cell replication [32, 33]. Moreover, for detection of complex DSB, e.g., induced by high LET irradiation, large foci size, delayed repair kinetics and the molecular composition of the foci (recruitment of different DNA-repair pathways) are used as indirect surrogates [32, 34]. Our data show that Cell-Fit-HD bio-detectors could be successfully employed for spatial correlation between particle tracks and radiation induced damage sites. However, an unambiguous definition for complex DSB e.g. by specific molecular marker or spatio-temporal characteristics remain elusive. Our study indicates an increase of RIF size as a function of time and LET, and a significant linear correlation between RIF-size and cellular survival. Intriguingly, the quantity of both initial- and residual- RIF well correlated with survival fraction as the reference RBE readout. However, residual RIF showed a more steep and robust correlation with cell survival (0.97) compared to initial RIF (0.92).

Together, these data underscore the value of RIF assessment in radiobiological dosimetry, in particular when QC of different radiation sources is performed. The discrepancies found in previous reports in favor or against determination of RIF as biodosimeters may in part rely on the large genetic intra- and intertumoral heterogeneity precluding generation of reproducible and robust biodosimeter, as well as formation of DNA repair centers [35]. In our study A549 human lung cancer cells were selected based on their very low background staining for classical RIF marker (γ-H2AX). Further, these cells fulfilled our stringent criteria for reproducibility and robust RIF induction, spatio-temporal kinetic and enumeration. We hypothesize that definition of a consensus set of cell lines will be central to assure a reliable benchmark for radiobiological QC of the growing number of particle therapy facilities.

The correlation of foci formation/area and clonogenic survival as function of LETd opens the possibility of extending the MC predictions incorporating a calculation scheme for foci as a function of particle energy and dose level. This may turn in a microscopic surrogate for the biological effect verification for advanced MC-based treatment planning system (TPS) calculations. To overcome current limitation of our approach for simulating DNA damage as the consequence of the complex mixed radiation field created in clinical beams a database of single strand breaks (SSB) and DSB as function of particle type and energy are needed. Hence, we seek to develop such a biological database using both, analytical approaches such as applying amorphous track structure codes as well as specific MC codes. This would allow the investigation of the spatial distribution of the DSB. In regards to the temporal study of the complex DSB, a model taking into account the repair dynamic as function of DSB complexity needs to be implemented. Together, the present work builds a solid foundation to assess biophysical beam quality in clinical settings for the growing number of different radiation sources utilized in the particle facilities.

## MATERIALS AND METHODS

### Radiotherapy plans

Alternative dose distribution plans were calculated with commercial treatment planning systems (Syngo RT planning system of Siemens Healthcare, Germany, for protons and carbon ions and VMAT plan with Oncentra planning system by Elekta, Sweden, for photon therapy). Syngo applies a RBE of 1.1 for proton [36], whereas for carbon ions the LEM-I biophysical model is applied clinically at HIT. The proton and carbon ion calculations have been performed with our clinical TPS applying. The treatment plans have been optimized for the PTV as a target to cover the 100% of the CTV with at least the 95% of the planned dose (37.5 Gy (RBE)). For carbon ions, the prescribed dose was 3 fractions of 8.25 Gy median physical dose corresponding to 12.5 Gy (RBE). The total dose of 37.5 Gy (RBE) in three fractions was calculated based on LEM-1. This is a conservative neurotoxicity centric RBE calculation that was successfully employed at GSI and is current clinical practice at HIT.

The carbon ion plans have been calculated using pencil beams having initial full-width half maximum (FWHM) in air of about 6 mm (FWHMxy) spaced laterally by 2 mm (stepxy). The water equivalent distance in depth between two consecutive energy slices is 3 mm (zslice). The single field enters with a gantry angle of 0° while for the two fields optimization gantry angles of 10° and 345° have been chosen. The two fields proton plan has been calculated using FWHMxy of about 8 mm, stepxy = 3 mm and zslice = 2 mm. The chosen gantry angles were 10° and 345°. The applied settings are consistent with the typical treatment planning parameters applied at HIT. For photons, a VMAT plan has been calculated with a single arc with angles (178.0–318.0) with 56 control points.

### Cell culture

Human alveolar adenocarcinoma cell line A549 (ATCC, Manassas, VA, USA), were grown in complete growth medium, consisting of Dulbecco's Modified Eagle Medium (DMEM, ATCC) supplemented with 10% heat-inactivated Fetal Bovine Serum (FBS, Millipore, Germany), 2 mM glutamine and 1% Penicillin/Streptomycin (Gibco, Germany) at 37°C at 5% CO_2_ atmosphere.

### Clonogenic survival assay

Clonogenic survival assay of A549 cells was performed as in previously described protocol [37]. Cells were seeded in 6-well plates (triplicates) or in 25 cm^2^ flasks (triplicates) and incubated at 37°C at 5% CO_2._ After attachment, cells were irradiated with different irradiation doses. Non-irradiated cells were used as control. After colonies formation, cells were fixed with 75% methanol and 25% acetic acid for 10 minutes at room temperature and labeled with 0.1% crystal violet for 15 min. Colonies containing more than 50 cells were counted as survivors.

### Irradiation treatment and calculation procedure

Photon irradiation was performed using a linear accelerator (LINAC, 6 MV, Artist, Siemens, Germany) at German Cancer Research Center (DKFZ). Particle irradiations were performed at HIT with the raster-scanning technique [38]. For irradiation with proton, helium-, carbon- and oxygen ion beams, cells were positioned in the middle of a 1 cm wide SOBP centered at about 3.5 cm water-equivalent depth. Plans have been optimized applying a research treatment planning system (TPS) available at HIT [39].

The planned dose levels were: 0.25 Gy, 1 Gy, 2 Gy and 4 Gy (dose rate ~0.5 Gy/min). MC forward calculations by means of the MC code FLUKA [40], similarly as in Bauer et al. 2014 [41], have been performed reproducing both measured dose-depth distributions and cell survival data. The latter has been achieved interfacing the MC advanced calculation capability of the mixed radiation field [26] with the HIT re-implementation of the GSI Local Effect Model (LEM, version IV [27, 42]). The input parameters of the LEM αx and βx (see Table 1), i.e. the linear-quadratic (LQ) model [19] parameters have been obtained fitting the photons cell survival experimental data using an in-house tool based on Minuit package available in ROOT [43]. The LQ parameters for the four charged particle irradiations have been also calculated and reported in the Table 1. The only free parameter for LEM-based calculations, Dt, has been adjusted matching the experimental data resulting in a value of 15 Gy. It represents the transition dose at the which the survival curve for photon is assumed to have an exponential shape with maximum slope equals to Smax = αx + βxDt. Other input parameters have been fixed as reported in previous work [27].

Dose measurements were performed using a water phantom (MP3-P, PTW, Germany) typically applied for patient plan verification [44]. Dose values can be measured simultaneously at 24 positions using 24 ionization chambers (ICs, PinPoint^®^ Ionization Chambers, 0.03 cm^3^, PTW) [4]. Several positions of the 24 ICs block have been chosen in order to measure accurately the SOBP along the entire penetration depth. The minimum measurable depth results from geometrical constraints of the experimental apparatus. Self-developing Gafchromic^®^ EBT2 films by International Specialty Products (ISP) (Wayne, NJ, USA), have been used to access lateral intensity distributions, where the 6-well plates have been located. The films have been digitalized using the Dosimetry Pro Advantage (RED) scanner (Vidar Systems Corporation, Herndon, VA, USA). A scan resolution of 300 dots per inch (dpi) and a color depth of 16 bits per channel were used. Neither filters nor dose calibration has been applied.

### Immunocytochemistry staining

Irradiated and control cells were fixed at different time points after irradiation using cold 70% ethanol and stored at −20°C overnight (or longer) before labeling. γ-H2AX labeling was performed using primary anti-γ-H2AX antibody (1:100, Cell Biolabs, San Diego, CA, USA) and secondary Alexa 488-conjugated anti-mouse secondary antibody (1:600, Life Technologies, Germany). Cells were washed in PBS and mounted with ProLong^®^ Gold antifade reagent with DAPI (Life Technologies).

### γ-H2AX foci analysis

Cells were seeded on glass cover slips, positioned parallel or perpendicular to a beam path and irradiated with different irradiation qualities at 1 Gy physical dose. This was followed by fixation and immuno-staining. Parallel beam irradiation was used for depicting the foci formation along the particle tracks. Images of the γ-H2AX foci and nuclei within irradiated cells were acquired sequentially with the motorized FV1000 confocal microscope (Olympus, Germany) controlled by the FluoView software and equipped with an Argon 488 nm and a UV diode 405 nm laser and reflected light photomultiplier tubes (PMTs), using the 60×/1.35 UPlanSApo objective. The 12-bit image stacks of on average 25 slices with the 0.3 μm distance between 512 × 512 pixel frames and 68 × 68 nm^2^ pixel size were acquired by keeping all the settings on the microscope constant. The stacks were further processed and 3D visualized by the Surpass module with the maximum intensity projection in the Imaris 7.7 software using the linear LUT and covering the full range of the data. For γ-H2AX foci quantification it was necessary to obtain time efficient information on a large cell population. Therefore, the 12-bit Z-stacks of 5 slices with the 2 μm distance between 1344 × 1024 pixel frames and 322 nm pixel size were acquired with constant microscope settings to visualize foci and nuclei with the motorized Olympus IX81 microscope controlled by the Xcellence software and equipped with the MT20 illumination system, GFP and DAPI HC-Filtersets, and Hamamatsu Orca-ER CCD camera (Hamamatsu, Japan), using the 20×/0.75 UPlanSApo (Olympus) objective. Foci scoring and analysis were performed for single stack taken in the central regions of the nuclei, using an ImageJ (http://rsbweb.nih.gov/ij) macro developed at DKFZ Light Microscopy Core Facility. All the images are processed with constant settings using ImageJ's Rolling ball background subtraction and z-projection and displayed with pseudo-color adjusted linearly the same for individual channels on merged images. Nuclei showing pan-γ-H2AX staining and apoptotic features were excluded from the analysis. For each nucleus, the number of individual foci within the nucleus was scored and analyzed (at least 100 nuclei per sample per condition). Foci number of control cells (non-irradiated) was subtracted from the corresponding foci number of irradiated cells. Control cells were characterized by low number of background foci (^~^0.16 per nucleus).

### Nuclear track detection and γ-H2AX tracks morphology

For particle traversals and γ-H2AX foci morphology analysis we utilized Cell-Fit-HD as previously described [11, 21]. Cell-Fit-HD allows assigning single ion traversals to corresponding subcellular damage sites in clinical ion beams. Briefly, cells (biological compartment) were cultured on the polished surface of FNTD (physical compartment, *Landauer* Inc, USA) [21]. After the cells formed a monolayer, Cell-Fit-HDs were placed in a 24 multiwell plate and irradiated perpendicularly with respect to the incident ion beam. The backside of the FNTD without cell coating was facing the incident ion beam. The cell layer was placed in themed SOBP (width = 1 cm, planned dose = 0.25 Gy) of carbon (oxygen) ion beam. The total MC calculated fluence (in terms of particle traversals per area) for carbon ion and oxygen ions beam considering primary beam and secondary heavy charged particles was about 3.8E + 06 particles/cm^2^ and 2.8E + 06 paritcles/cm^2^, respectively.

### Nuclear track statistics

#### Cell-Fit-HD read-out

The Cell-Fit-HD was imaged sequentially using the LSM710, Confocor 3 confocal laser scanning microscope (Zeiss, Germany) equipped with a piezo stage, 63×/1.40 NA, 40×/1.30 NA oil objectives, PMT and avalanche photo diodes (APD). The protocols used were as previously described in [21]. For the intranuclear hit statistics the FNTD was read-out with a 633 nm HeNe laser line, 40× oil objective, dichroic beam splitter MBS 488/561/633 nm, 655 nm long-pass filter, APD emission detection (photon counting mode). Following parameters were used for: oxygen ions (relative laser power *p* = 7%, pixel dwell time τ = 7.2 μs, number of rescans *R* =1), carbon ions (*p* = 10%, τ = 7.2 μs *R* = 1,). The pinhole was set to 1AU. An imaging plane of 212.55 × 212.55 μm^2^ (1800 × 1800 pixels²) at approximately 3 μm depth measured from the FNTD surface was recorded. Subsequently, the cell layer was recorded (pixel settings were equal to FNTD read-out). The cell layer stack covered a range of approximately 10 μm with an axial step size of Δ z = 0.5 μm. The cell layer was imaged with a 405-nm diode laser (*p* = 0.5%) for DAPI and with a 488-nm Argon laser line (*p* = 2%) for Alexa488 (γ-H2AX). Both channels were imaged with τ = 7.2 *R* =1μs,). For DAPI and Alexa488 a MBS 405 nm and MBS 488/561/633 nm and PMT detection (detection windows: 410–483 nm and 493–630 nm, 1AU) were used, respectively. For the spatial correlation of γ-H2AX foci and single ion traversals similar imaging parameters were used (63× oil objective).

### Intranuclear ion hits

The number of primary particles traversing a cell nucleus was accessed for the perpendicular irradiation setup. To access the number of intranuclear hits, all track spot centers were projected onto the cross sectional area of the cell nuclei. The cross sectional area was gained by image segmentation (marker based watershed segmentation) of the maximum intensity projection of the DAPI channel. Ion track spots were detected by thresholding of the top-hat filtered FNTD raw image data (filter kernel: disk of radius 3 pixel). The maximum intensity of all track spots identified was measured and converted into count rate. A histogram-based threshold of the APD signal was then used to distinguish between primary ions and fragments. It was set to 4.4 MHz (oxygen) and 5.3 MHz (carbon). Heavy projectile-like fragments, i.e. secondary particles produced in the nuclear interactions having similar characteristics as the primary beam cannot be distinguish from the beam and are included in the performed MC simulations.

### Statistical analysis

The data of different groups were compared using two sided analysis of variance (ANOVA) test. *P values* less than 0.05 (95% confidence interval) were considered as statistically significant. ANOVA, linear regression, and Pearson *R*^2^ correlation calculations were performed using GraphPad Prism software.

## SUPPLEMENTARY MATERIALS FIGURES


