# Supplementary material for: Next generation multi-scale biophysical characterization of high precision cancer particle radiotherapy using clinical proton, helium-, carbon- and oxygen ion beams
Source: Oncotarget. 2016 Aug 1;7(35):56676–89. doi: 10.18632/oncotarget.10996 (PMC5302944; doi:10.18632/oncotarget.10996)
Supplement: Supplementary file 1 [file oncotarget-07-56676-s001.pdf]

Next generation multi-scale biophysical characterization of high precision cancer particle radiotherapy using clinical proton, helium-, carbon- and oxygen ion beams

Supplementary Materials

SUPPLEMENTARY METHODS

Intracellular reactive oxygen species measurement

For reactive oxygen species (ROS) detection in A549 cells CM-H2DCFDA probe (Molecular Probes/ Thermo Fisher Scientific, USA) has been used. Briefly, cells were seeded in T25 flasks or 6 well plates and allowed to attach overnight. After attachment, cells were irradiated with 1 Gy photons, ^{1}H, ^{4}He, ^{12}C, ^{16}O. Non irradiated cells were used as a control. After irradiation cells were cultured for next 48 h. At this point cells were collected and labeled with 5 µM CM-H2DCFDA (37°C for 20 min). This was followed by extensive washing with PBS. After labeling, cells were immediately analyzed at FACS Calibur flow cytometer. Analysis was performed using CellQuest software.

Caspase 3/7 mediated apoptosis

Apoptosis was assessed by measuring the number of cells positive for activated caspase 3/7 at different time points. For this purpose cells were first seeded in 96-well plates and allowed to attach overnight. Shortly before irradiation (1 Gy photons, ^{1}H, ^{4}He, ^{12}C, ^{16}O, non-irradiated cells were used as a control) cells were labeled with CellPlayer™ Kinetic Caspase-3/7 Apoptosis Assay Reagent (Essen Bioscience,USA), as described in manufacturers protocol. Briefly, reagent (5 µM) was added to the cell media, and cells were placed in the incubator. No washing steps were required. After labeling, at indicated time points, four images per well (2 × 2 tiles region stitched using Zeiss ZEN blue software) were taken using motorized Zeiss Cell Observer. Z1 microscope equipped with the LED Colibri excitation source, 62 HE filterset, and MRm CCD AxioCam. Images were further processed automatically with the same settings using ImageJ macro developed specifically for this purpose. Images of cells stained with the Apoptosis Assay Reagent were background subtracted using Rolling ball radius algorithm, images were further filtered using Gaussian Blur, segmented using Find Maxima above the Threshold, and analyzed by the Analyze Particles ImageJ tool. Number of apoptotic cells (positive stained cells, green fluorescence) was compared to the number of total cells in each stitched image in each sample.

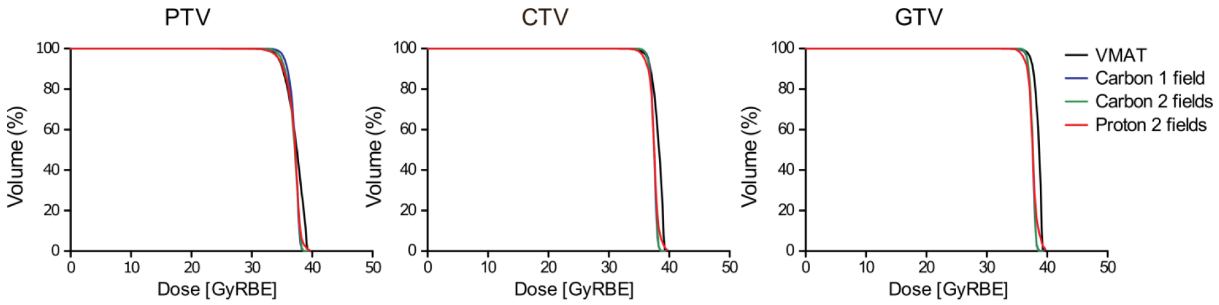

Supplementary Figure S1: Dose-volume histograms for three main volumes in radiotherapy planning. Figure represents dose-volume histograms for comparing three different radiotherapy plan simulations in a lung cancer patient: Volumetric Intensity Modulated Arc Therapy (VMAT), proton therapy with two fields, carbon therapy with one field and carbon therapy with two fields (Figure 1 in the main text). Dose-volume distribution is presented for three main target volumes: planning target volume (PTV) takes into account possible deviations in planning and treatment administration and it ensures that the organ at risk does not receive hazardous dose, while providing delivery of prescribed treatment dose to the clinical target volume (CTV). CTV takes into account gross tumor volume (GTV) and an additional margin for a possible tumor distribution which cannot be precisely imaged. GTV defines the position and size of gross tumor that can be accurately defined using high quality imaging techniques.

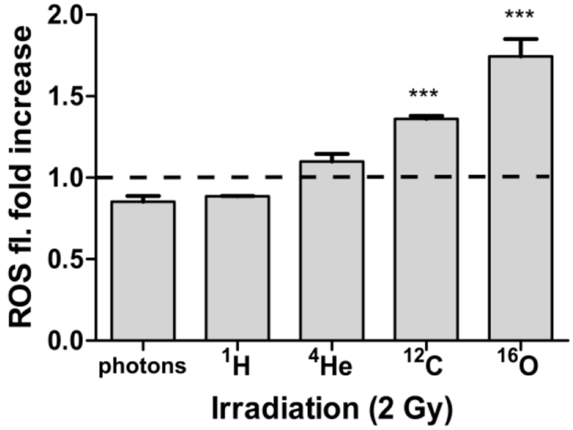

Supplementary Figure S2: Intracellular ROS accumulation. ROS levels in irradiated cells 48 h post – irradiation (2 Gy). While photon and ^{1}H-beams did not affect the accumulation of late ROS, intracellular ROS levels increased with an increase of LET (^{4}He < ^{12}C < ^{16}O). Data are expressed as normalized values of the mean fluorescence intensity (MFI) of irradiated samples to the MFI of the control (1.0, dashed line). Bars represent the means and standard errors. **p* < 0.05, ****p* < 0.005, one way ANOVA with Bonferroni correction.

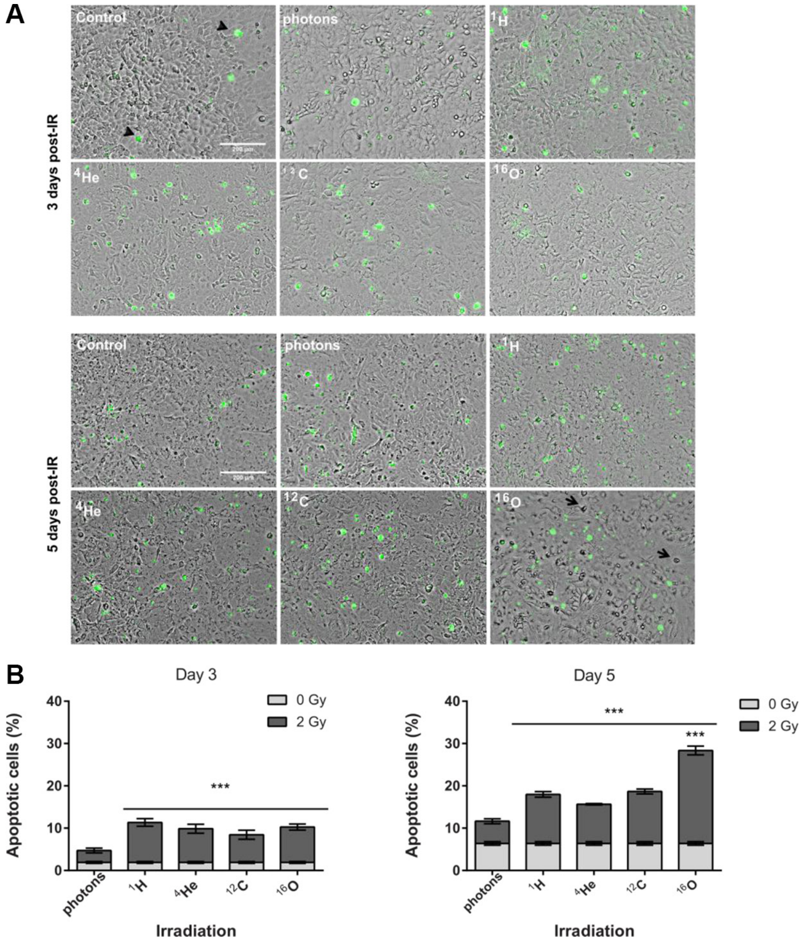

Supplementary Figure S3: Caspase 3/7 mediated apoptosis. (**A**) Representative images of irradiated A549 cells at day 3 and day 5 post-irradiation (2 Gy). Arrowheads point to apoptotic cells, i.e., activation of Caspase 3/7 leads to the nuclear localization of green fluorescence. Arrows indicate dead cell debris. Scale bar = 200 µm. (**B**) Mean percentage of apoptotic cells (compared to the total cell number) and SEM were shown at day 3 and day 5 post- irradiation (*n* = 4, **p* < 0.05, ***p* < 0.01, ****p* < 0.005). Radiation-induced apoptosis was observed in only up to ~10% and 30% at day 3 and day 5 post-irradiation, respectively. Despites the relatively low fraction of A549 cells undergoing apoptosis, particle irradiation induced significantly higher caspase 3/7 mediated apoptosis when compared to photon irradiation treatment. However the was no significant difference in the level of apoptosis induction between different particle qualities at Day 3 post irradiation. In ^{16}O-irradiated samples, the level of apoptosis was at the highest at day 5 post-irradiation.
